# [Retracted] Upregulation of microRNA‑181b inhibits CCL18‑induced breast cancer cell metastasis and invasion via the NF‑κB signaling pathway

**DOI:** 10.3892/ol.2024.14401

**Published:** 2024-04-15

**Authors:** Lei Wang, Yu-Xia Wang, Li-Ping Chen, Ming-Li Ji

Oncol Lett 12: 4411–4418, 2016; DOI: 10.3892/ol.2016.5230

Following the publication of this paper, it was drawn to the Editor's attention by a concerned reader that the cell migration and invasion assay data featured in Fig. 2A and B on p. 4414 had already been published in an article in the journal *PLoS One* (doi.org/10.1371/journal.pone.0117518) written by different authors at different research institutes. Owing to the fact that the contentious data in the above article had already been published prior to its submission to *Oncology Letters*, the Editor has decided that this paper should be retracted from the Journal. After having been in contact with the authors, they accepted the decision to retract the paper. The Editor apologizes to the readership for any inconvenience caused.

